# Redescription and Phylogenetic Analysis of the Mandible of an Enigmatic Pennsylvanian (Late Carboniferous) Tetrapod from Nova Scotia, and the Lability of Meckelian Jaw Ossification

**DOI:** 10.1371/journal.pone.0109717

**Published:** 2014-10-07

**Authors:** Roland B. Sookias, Christine Böhmer, Jennifer A. Clack

**Affiliations:** 1 School of Geography, Earth and Environmental Sciences, University of Birmingham, Edgbaston, Birmingham, United Kingdom; 2 GeoBio-Center, Ludwig-Maximilians-Universität München, München, Germany; 3 Dept. für Geo- und Umweltwissenschaften, Ludwig-Maximilians-Universität München, München, Germany; 4 Bayerische Staatssammlung für Paläontologie und Geologie München, München, Germany; 5 University Museum of Zoology Cambridge, Cambridge, United Kingdom; Raymond M. Alf Museum of Paleontology, United States of America

## Abstract

The lower jaw of an unidentified Pennsylvanian (Late Carboniferous) tetrapod from Nova Scotia – the “Parrsboro jaw”- is redescribed in the light of recent tetrapod discoveries and work on evolution of tetrapod mandibular morphology and placed for the first time in a numerical cladistics analysis. All phylogenetic analyses place the jaw in a crownward polytomy of baphetids, temnospondyls, and embolomeres. Several features resemble baphetids and temnospondyls including dermal ornamentation, absence of coronoid teeth, and presence of coronoid shagreen. Dentary dentition is most similar to *Baphetes*. An adsymphysial toothplate may not preclude temnospondyl affinity. An apparent large exomeckelian fenestra, with the dorsal foraminal margins formed by an unossified element, echoes the morphology of the stem tetrapod *Sigournea* and is unusually primitive given the other features of the jaw. The jaw may thus provide an example of an intermediate stage in Meckelian element evolution.

## Introduction

The lower jaw of primitive tetrapods and tetrapodomorph fishes is composed of a large number of separate ossified elements, some endochondral and some dermal. The lower jaw is thus a complex structure and thereby likely to yield phylogenetic information. Despite this, the lower jaw of primitive tetrapods was largely ignored in phylogenetic studies until the last two decades, with the exception of the work of Gross [1]. During the 1990s our understanding of the phylogenetic importance of the tetrapod jaw has improved greatly owing to discovery of new material and reassessment of old specimens [2], [3]. Although many tetrapod apomorphies had been previously identified [4], additional key characters (e.g. degree of exposure of Meckelian bone; form of coronoid and adsymphysial dentition) differentiating tetrapod and tetrapodomorph fish ( =  “osteolepiform”) jaws have been recognized subsequently. This has allowed the identification of several specimens previously identified as fishes as stem tetrapods (e.g. [3]). The review of Ahlberg and Clack [2] identified many of the key changes seen in the lower jaws of tetrapods that distinguish them from tetrapodomorph fishes, and has been augmented by the work of Bolt and Lombard [5] and Lombard and Bolt [6]. Such placement of lower jaw characters in a cladistic framework has exposed the complexity of the sequence of character change in the tetrapod lineage, with tetrapod characters acquired individually along the tetrapod stem, rather than necessarily being diagnostic of crown Tetrapoda. Use of mandibular characters in particular has been successful in improving resolution of tetrapod phylogeny, and because mandibles are relatively durable and often remain articulated [5], further mandibular analyses are likely to bear fruit.

The natural mould of the left half of a tetrapod lower jaw –NSM 987GH65.1, henceforth the Parrsboro jaw - was discovered in 1987 west of Parrsboro, Nova Scotia, close to the town of Diligent River [7]. The mould was in sandstone of the Parrsboro Formation, assigned to the lowermost Pennsylvanian (formerly Westphalian A) by macrophyte and microspore fossils [8]. This formation has provided excellent early tetrapod ichnofossils [8], but has yielded only one other example of tetrapod skeletal material, attributed to *Dendrerpeton* [9]. The jaw was initially described as an indeterminate tetrapod [7] based on its possession of a number of characters deemed to be “autapomorphic for Tetrapoda” including lack of coronoid tusks/replacement pits and an open sensory canal. At the time of description, the evolution of mandibular structure in early tetrapods was relatively poorly understood - most characters discussed were deemed simply “plesiomorphic for tetrapods”, with the complexity and sequence of character change and acquisition not elucidated. Here the material is reanalyzed in the light of Ahlberg and Clack's [2] work and subsequent descriptions of the mandible of Carboniferous tetrapods including *Greererpeton* [5], *Whatcheeria* [6], *Sigournea* [10], and *Ymeria* [11]. Along with a brief redescription of the specimen, a phylogenetic analysis is undertaken.

## Materials and Methods

**Figure 1:**
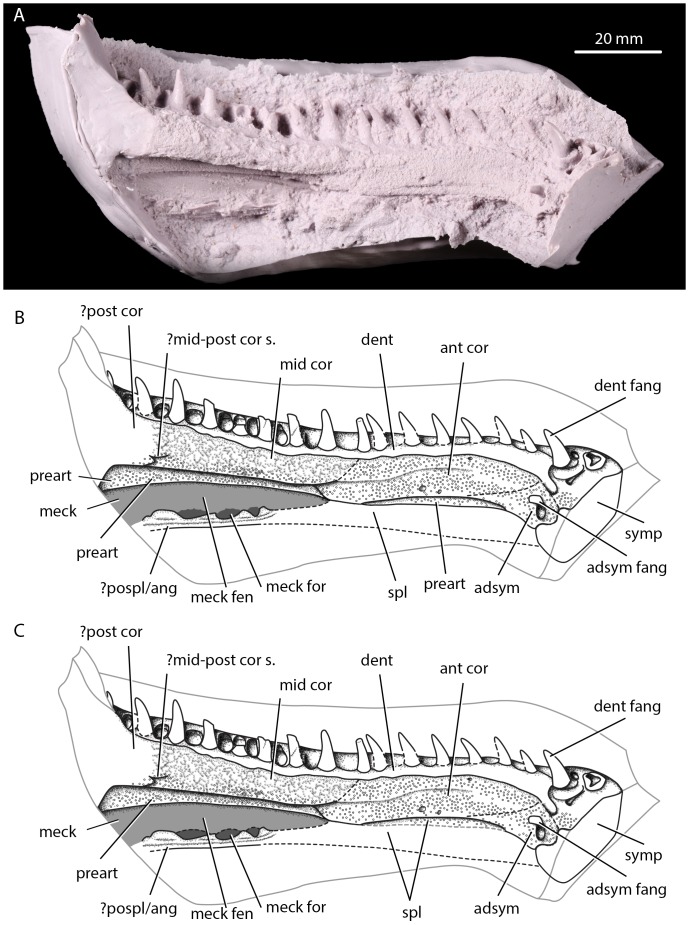
Anterior portion of the Parrsboro jaw, NSM 987GH65.1, in medial view. A, photograph of anterior part of jaw natural mould peel loaned to JAC in medial view; B, interpretative line drawing of anterior part of jaw in medial view; C, alternative interpretative line drawing of anterior jaw in medial view, with denticulated element below anterior coronoid interpreted as splenial rather than prearticular. adsym, adsymphyseal toothplate; adsym fang, adsymphyseal fang; ant cor, anterior coronoid; dent, dentary; dent fang, dentary fang; meck for, Meckelian foramina; meck, unossified Meckelian element; mid cor, middle coronoid; ?mid-post cor s., possible suture between middle and posterior coronoids; pospl, postsplenial; ?post cor, possible posterior coronoid; preart, prearticular; spl, splenial. Hollow circular stippling indicates denticulation.

**Figure 2:**
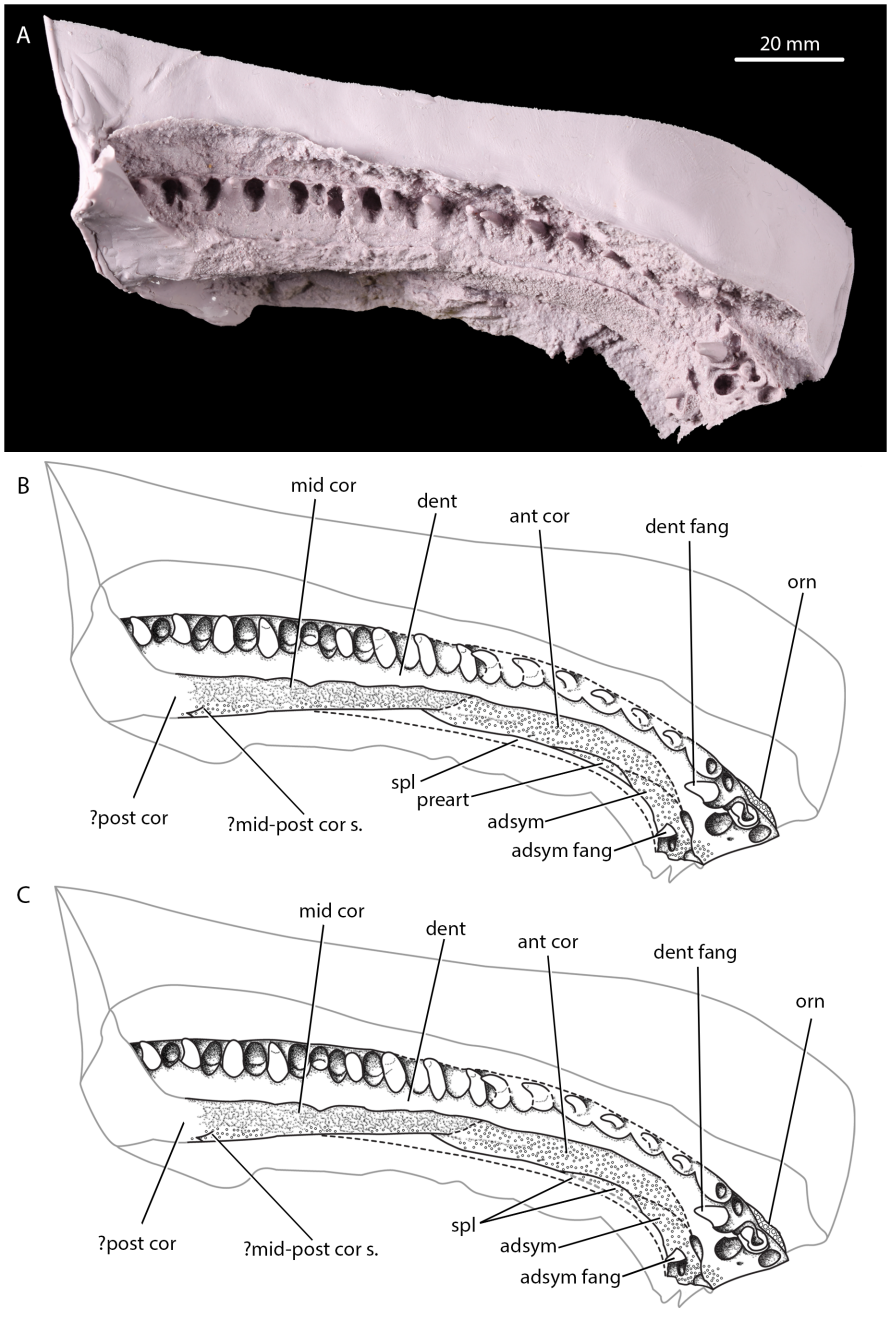
Anterior portion of the Parrsboro jaw, NSM 987GH65.1, in dorsal view. A, photograph of peel of anterior of natural mould of jaw loaned to JAC in dorsal view; B, interpretative line drawing of anterior of jaw in dorsal view; C, alternative interpretative line drawing of anterior jaw in dorsal view, with denticulated element below anterior coronoid interpreted as splenial rather than prearticular. adsym, adsymphyseal toothplate; adsym fang, adsymphyseal fang; ant cor, anterior coronoid; dent, dentary; dent fang, dentary fang; mid cor, middle coronoid; ?mid-post cor s., possible suture between middle and posterior coronoids; orn, ornamentation; ?post cor, possible posterior coronoid; preart, prearticular; spl, splenial. Hollow circular stippling indicates denticulation.

This description of the Parrsboro jaw, like that of Godfrey and Homes [7], is based on Nova Scotia Museum specimen NSM 987GH65.1 - a latex peel of a natural mould (see [12], [13] for techniques used), a copy of which (see Figs. 1A, 2A, and supplementary laser scan video Video S1) was provided to JAC by the Nova Scotia Museum. The peel consists of the left hand side anterior mesial face of the mandible from the mandibular symphysis to just posterior to the most posterior tooth. The posterior portion of the jaw was, however, not included in the peel available to us, and the figures and description of Godfrey and Holmes [7] were used to assess more posterior characters. A photograph of a mould of part of the lateral surface ([7], fig. 2B) was used for assessing lateral ornament pattern. A reasonable amount of anatomical detail is visible, though the matrix is coarse-grained sandstone and in some places details are difficult to discern. We did not examine the posterior part of the jaw as it was not available to us, and [7] was relied upon to score it.

A phylogenetic analysis was carried out in TNT v. 1.1 ([14], [15]). A tree bisection-reconnection (TBR) heuristic search saving 1000 Wagner tree replicates to the RAM (with random addition sequence – RAS) was carried out, followed by a branch and bound search based on these trees. A modified version of the matrix of Clack et al. [11] was used (see Supplementary Information for list of characters and matrix) as it includes most key stem and basal crown group tetrapods and a large number of characters (115) including cranial, mandibular, and postcranial characters. The terminal ANSP 21530 was removed and the taxa *Sigournea, Caerorhachis* and *Occidens* were added to create a more representative sample of more crownward forms. Due to uncertainty with regards to identification of some of the elements (see different interpretations in Fig. 1), two separate versions of this matrix (matrices 1 and 2, corresponding to Figs. 1A and 2A and Figs. 1B and 2B respectively) were scored and analysed. *Eusthenopteron* was used as the outgroup. Analyses were carried out with all characters unordered, with characters 33, 54, 70, 72, 108, 109 and 115 ordered, and including and excluding the most incomplete taxa (*Metaxygnathus* and *Densignathus*), following [11]. Each analysis was carried out using both matrix 1 and 2 (see Table 1) and with all characters and only mandibular characters. Standard bootstrap values were calculated in TNT.

**Table 1:** Table summarizing results of all phylogenetic analyses carried out for the Parrsboro jaw, NSM 987GH65.1.

Analysis number	Matrix used	Incomplete taxa excluded?	Ordered characters?	Mandibular characters only?	Number of MPTs	Number of steps	CI	RI
1.1	1	No	No	No	224	271	0.513	0.680
1.2	1	Yes	No	No	84	266	0.523	0.692
1.3	1	No	Yes	No	112	274	0.507	0.688
1.4	1	Yes	Yes	No	24	269	0.517	0.700
1.5	1	No	No	Yes	2137	106	0.547	0.750
1.6	1	Yes	No	Yes	1951	103	0.563	0.766
1.7	1	No	Yes	Yes	2137	106	0.547	0.750
1.8	1	Yes	Yes	Yes	1951	103	0.563	0.766
2.1	2	No	No	No	96	269	0.517	0.682
2.2	2	Yes	No	No	36	264	0.527	0.694
2.3	2	No	Yes	No	56	272	0.511	0.691
2.4	2	Yes	Yes	No	12	267	0.521	0.702
2.5	2	No	No	Yes	1696	105	0.552	0.751
2.6	2	Yes	No	Yes	1540	102	0.569	0.767
2.7	2	No	Yes	Yes	1696	105	0.552	0.751
2.8	2	Yes	Yes	Yes	1540	102	0.569	0.767

Matrix 1 interprets the denticulated element below the anterior coronoid as a continuation of the prearticular (Figs. 1B, 2B) whereas matrix 2 interprets this element as part of the splenial (Figs. 1C, 2C). On each matrix, separate analyses were carried out with all taxa used in Clack et al. (2012),with highly incomplete taxa (*Metaxygnathus* and *Densignathus*) pruned, with only mandibular and with all characters, and with both all characters unordered and with characters 33, 54, 70, 72, 108, 109 and 115 ordered, following Clack et al. (2012). This yielded a total of sixteen separate analyses.

MPTs  =  most parsimonious trees; CI  =  consistency index; RI  =  retention index.

No permits were required for the described study, which complied with all relevant regulations.

## Results

### Systematic palaeontology and description

OSTEICHTHYES Huxley 1880 [16]

TETRAPODOMORPHA Ahlberg 1991 [17]

TETRAPODA Haworth 1825 [18], *sensu* Goodrich 1930 [19]

#### Overall shape

The peel examined (i.e. the anterior portion of the natural mould) is 130 mm in length along a straight line from the mesial margin of the mandibular symphysis to the posteriormost part of the dentary. The jaw's full length as described by Godfrey and Holmes [7] is 192 mm. The mandible is 34 mm deep dorsoventrally at the deepest point, measured from the ventralmost to dorsalmost ossified elements (dentary and postsplenial respectively), and 4–5 mm deeper when measured to the tip of the tooth at the deepest point. Following the method of Clack [20] it has a depth/length ratio of 22.4%, similar to *Neopteroplax* [20].

#### Coronoids

**Figure 3:**
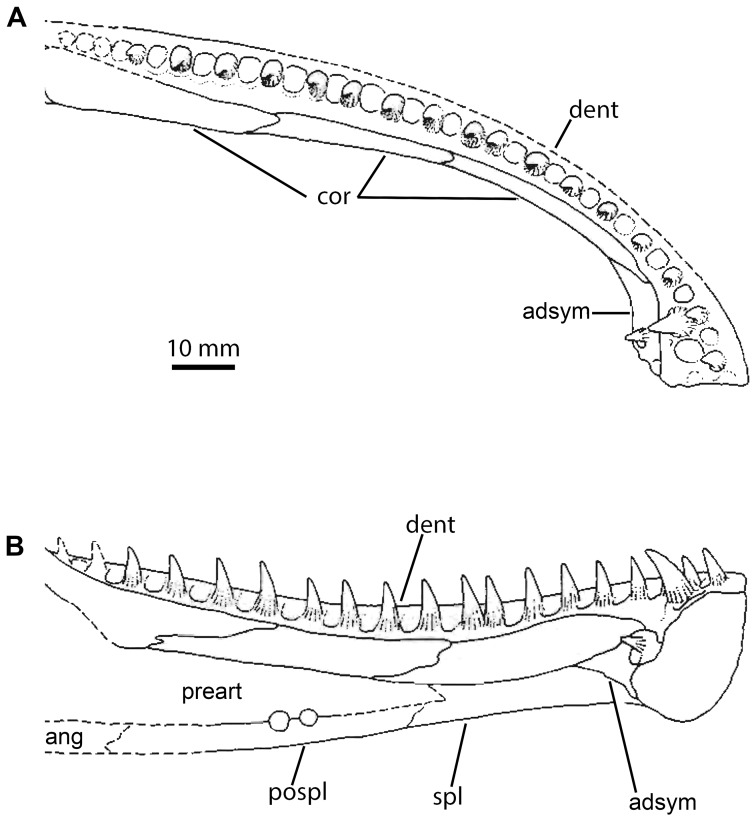
Interpretive line drawings of the anterior of the Parrsboro jaw, NSM 987GH65.1, modified from **fig. 1** of Godfrey and Holmes (1989). A, dorsal view; B, medial view. adsym, adsymphyseal tooth plate; ang, angular; cor, coronoid; dent, dentary; pospl, postsplenial; preart, prearticular.

The anterior and middle coronoids are visible. The exact path of the suture line between the anterior and middle coronoids is unclear, although it is seemingly not greatly interdigitating. Godfrey and Holmes ([7]; Fig. 3) indicate a suture between the middle and posterior coronoid. However, all but the ventralmost portion of this feature is difficult to discern, and it may not in fact represent a suture. The coronoids wholly lack dentition. This is also the case in all Carboniferous tetrapods except the whatcheeriids [6] and *Occidens* [21]; coronoid dentition is a primitive feature of stem tetrapods [2] and is lacked by baphetids [22], [23], temnospondyls [22], [24] and *Caerorhachis* [25] among others. Denticle shagreen covers all but the most posterodorsal portion of the anterior coronoid, the ventral portion of the middle coronoid, and what may be the anterior end of the posterior coronoid. The dorsal portion of the middle coronoid and the undenticulated portion of the anterior coronoid display irregular sculpting.

#### Adsymphysial toothplate and dentition

An adsymphysial toothplate is present – as indicated by Godfrey and Holmes [7]. The adsymphysial toothplate shows a single adsymphysial fang and denticle shagreen, but no other dentition. There are 17 teeth in the main dentary tooth row separated by alveoli. The statement of Godfrey and Holmes [7] that there is room for 35 teeth including empty alveoli in the dentary tooth row is thus reasonable. Four dentary teeth are partially or entirely broken, and the tips are missing from four others. An additional dentary fang is set in mesially from the main row. Ten replacement pits are visible - excluding those of the dentary and adsymphysial fangs which both possess a replacement pit mesial to their position - with the remaining pits obscured by matrix. The adsymphysial and dentary teeth are all of similar size and shape, pointed and posterodistally recurved.

#### Infradentaries and prearticular

**Figure 4:**
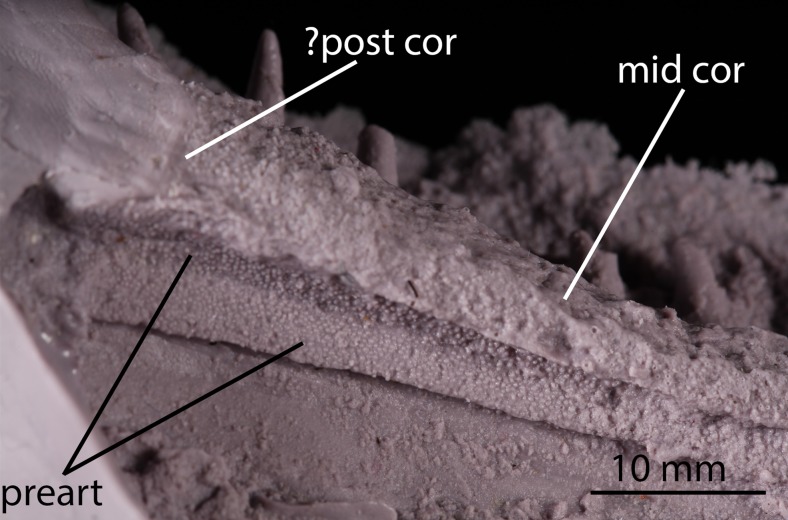
Close up of prearticular of the Parrsboro jaw, NSM 987GH65.1. The dorsal section of prearticular can be seen to have been displaced so that its medial surface faces ventromedially. mid cor, middle coronoid; ?post cor, possible posterior coronoid; preart, prearticular.

A denticulated area of bone ventral to the middle coronoid and dorsal to the unossified Meckelian element can be identified, based on position, as the prearticular. Prearticular denticulation is not uncommon, but is usually reduced to scattered patches in Carboniferous tetrapods except *Whatcheeria* [6] and *Crassigyrinus* [25] where (as in *Acanthostega* and *Ichthyostega*) it forms a well-defined dorsal band. In *Panderichthys* and *Eusthenopteron* there is no clear dorsal band, with the denticles instead gradually decreasing in density and ultimately disappearing ventrally [2]. Neither state appears to be present in the Parrsboro jaw. We thus coin a third state of the character based on this feature: fully denticulated. *Caerorhachis* shows a similar condition to the jaw in this regard; the lack of denticulation on the posterior half of the prearticular in the reconstruction of Ruta et al. [25] is due to this area not being preserved, with the anterior of the element being fully denticulated. The prearticular of the Parrsboro jaw appears to have been dorsoventrally compressed post mortem, so that the dorsalmost portion now lies horizontally beneath the middle coronoid (Fig. 4).

An element, either identifiable as the anterior of the prearticular or as the dorsal part of the splenial (see Figs. 1–2), is visible beneath the anterior coronoid. This element is fully and uniformly denticulated. Splenial denticulation is less common than prearticular denticulation, but does occur in early tetrapods [25]. Lack of preservation of the area where the prearticular-splenial suture would be expected (were the element the splenial) impedes identification of the element. A postsplenial forms the ventral edge of three exomeckelian foramina. As the prearticular lacks any downward protruberances which would have formed the dorsal portions of these fenestrae, it appears that the dorsal margins were instead formed by an unossified Meckelian element in life. The postsplenial lacks any denticulation. Preservation obscures any suture with the anterior prearticular/splenial area of ossification, and the assignment as a postsplenial is made based on position and lack of denticulation.

#### Posterior part of jaw

The jaw ramus posterior to the tooth row was not visible in the peel examined and the drawing and description of Godfrey and Holmes [7] was relied upon. The jaw appears to show an adductor crest morphology similar to *Caerorhachis* [25] or *Greererpeton* [5], with a low but noticeable, convexly curved crest with a maximum of convexity roughly level with the anteroposterior midpoint of the adductor fossa. In mesial view the lateral margin of the adductor fossa is, due to the adductor crest, convex and higher than the mesial margin, which is concave. A mesially projecting flange on the prearticular along the rim of the adductor fossa is described and figured as present. The posterior coronoid forms a small crest which formed the anterior of the adductor crest, but this contribution far less extensive than in *Pholiderpeton* [20].

#### Dermal ornamentation

Dermal ornamentation is preserved on the lateral side of the dentary just anterior to the dentary fang. The ornamentation is of a fine pit and ridge form. A larger section of ornament from the lateral surface was also preserved and is included as a photograph by Godfrey and Holmes [7]. It is also fairly regular pit and ridge ornamentation, as seen in temnospondyls, baphetids, colosteids and other taxa. Lateral line sulci cannot be identified.

### Phylogenetic analysis

**Figure 5:**
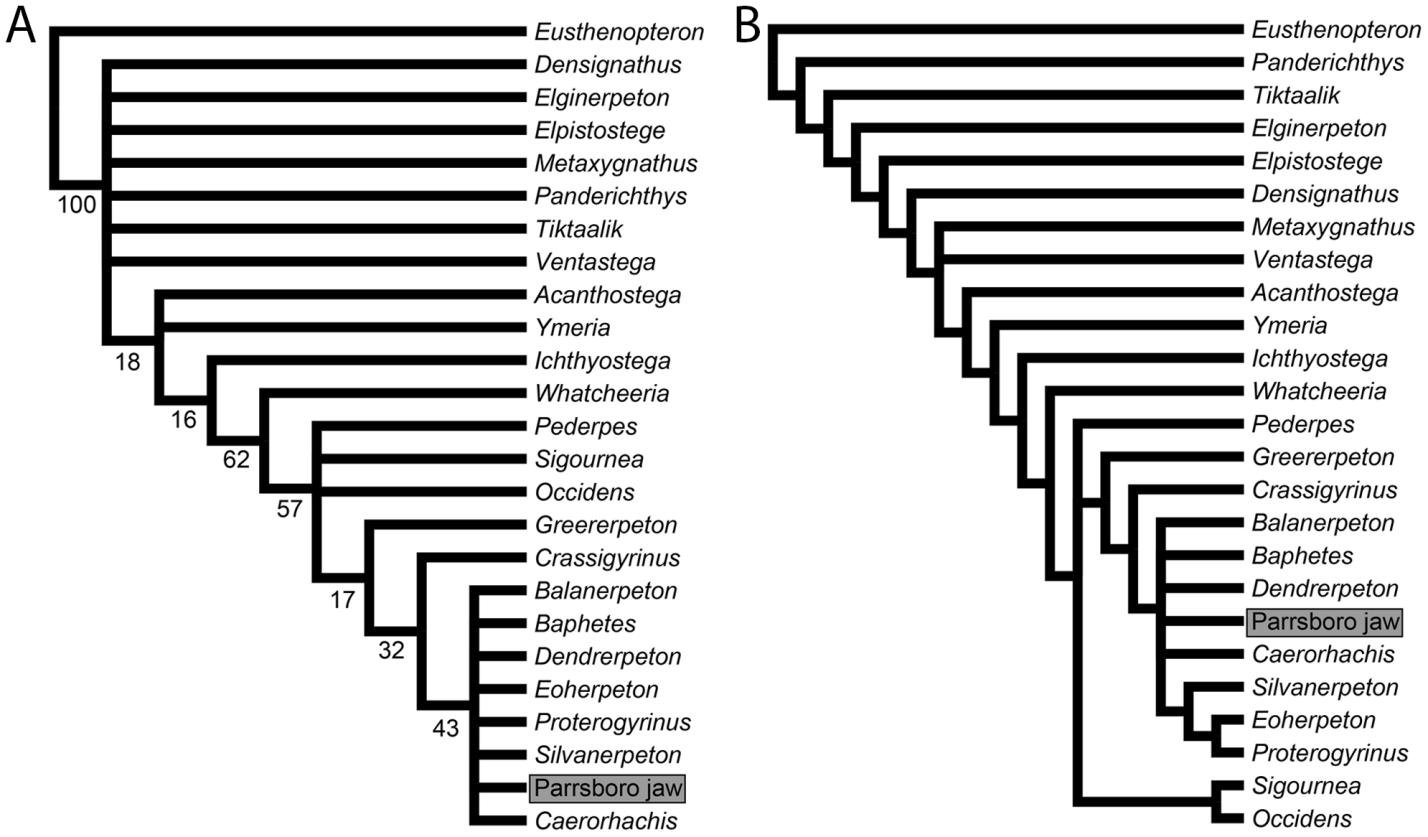
Phylogenetic position of the Parrsboro jaw, NSM 987GH65.1. The matrix used was based on the matrix of Clack et al. (2012). The analyses show correspond to Analysis 1.1 in Table 1, with the denticulated element below the anterior coronoid interpreted as a continuation of the prearticular (Figs. 1B, 2B), all characters unordered and no taxa excluded. A, strict consensus tree; B, 50 percent majority rule consensus tree. Numbers next to nodes are standard bootstrap values.

Numbers of most parsimonious trees (MPTs), tree lengths (i.e. minimum numbers of steps), and consistency and retention indices are given in Table 1. In all analyses using all characters, using both a strict and 50 percent majority rule consensus, the Parrsboro jaw was placed as part of a crownward polytomy with *Caerorhachis*, temnospondyls, baphetids and embolomeres (*Balanerpeton*, *Baphetes*, *Dendrerpeton*, *Eoherpeton*, *Proterogyrinus*, *Silvanerpeton*; Fig. 5). Bootstrap support for this clade was low (<45 for all analyses and <40 for all analyses using matrix 2; Fig. 5), reflecting the lack of resolution within the clade. An embolomere clade was resolved within the polytomy in strict consensus trees using matrix 2 but not matrix 1. Temnospondyl and embolomere + *Caerorhachis* clades were resolved within this polytomy in the majority rule trees, but the Parrsboro jaw remained in a polytomy with these clades and *Baphetes*. Part of the stem was also more resolved in the majority rule consensus trees. When mandibular characters alone were used, strict consensus trees were entirely unresolved but majority rule consensus trees placed the Parrsboro jaw either as the sister taxon to *Proterogyrinus*, in turn sister to *Eoherpeton* (using matrix 1), or in a polytomy with *Proterogyrinus* and *Eoherpeton*, sister to *Caerorhachis* (using matrix 2).

Of the characters scored for the Parrsboro jaw, in all analyses using the whole skeletal dataset, 63 (postsplenial with mesial lamina, no->yes), and 83 (adsymphysial plate dentition, organized dentition->shagreen or irregular tooth field) mapped as unambiguous synapomorphies of the clade bracketing the Parrsboro jaw; character 72 (coronoids: at least one fang pair, yes->no) mapped here in unordered analyses with matrix 2. In all analyses using the whole skeletal dataset character 75 (coronoids: organized tooth row, yes->no) mapped as a synapomorphy of the clade bracketing *Crassigyrinus* and the jaw. In all whole skeleton analyses, characters 59 (Meckelian fenestrae, dorsal margins formed by: prearticular->Meckelian bone), 63 (postspenial with mesial lamina, yes->no), 69 (prearticular with mesially projecting flange along adductor fossa, no->yes) and 86 (prearticular shagreen field, scattered or absent->gradually decreasing from dorsal to ventral) mapped as local autapomorphies of the jaw; 45 (coronoid contacts splenial, yes->no) and 60 (Meckelian fenestrae, much lower than adjacent prearticular->equal to or greater than adjacent prearticular) also mapped here using matrix 1, except in an ordered analysis excluding incomplete taxa. Using the mandibular dataset only, characters 42 (adductor fossa faces dorsally->mesially) and 45 (anterior coronoid contacts splenial, yes->no) mapped as autapomorphies of the jaw, though the strict consensus trees were polytomies.

## Discussion

Although the exact position of the Parrsboro jaw remains uncertain, it is unambiguously a stem or crown tetrapod, and crownward placement is plausible. Presence of an adsymphysial plate with true dentition as opposed to denticle shagreen is an unambiguous character of the tetrapod lineage [2], [3], differentiating stem tetrapod jaws from those of other sarcopterygians. Lack of exposure of Meckelian bone in precoronoid, intercoronoid and coronoid fossae, and the corresponding contact between the anterior coronoid and the splenial, is also an umabiguous apomorphy of the tetrapod lineage [3], and is displayed in the Parrsboro jaw. The jaw is distinguished from Devonian forms such as *Acanthostega* [2], *Densignathus* [26], and primitive Carboniferous tetrapods such as *Whatcheeria* [6], by absence of coronoid dentition. A trend in early tetrapod evolution is reduction of coronoid dentition [6], [2], and this places the jaw crownward of whatcheeriids and *Occidens* [21]. Like most embolomeres, temnospondyls and some baphetids, denticle shagreen covers the coronoids. The mesial direction of the adductor fossa, presence of a posterodorsal process on the posterior coronoid, and lack of an adsymphysial mesial foramen also make the jaw more similar to more crownward, Carboniferous forms (e.g. *Whatcheeria, Greererpeton*, *Sigournea*) and crown tetrapods than to Devonian taxa (e.g. *Acanthostega, Densignathus*), and the jaw possesses an adductor crest, a feature restricted to crown taxa and *Greererpeton*.

Despite its probable crownward position, the Parrsboro jaw appears to show the surprisingly plesiomorphic feature of an unossified Meckelian bone which forms the dorsal margins of the Meckelian foramina. Such an unossified Meckelian bone is absent in baphetids, temnospondyls and embolomeres [11]. This aspect of the morphology of the Parrsboro jaw is very similar to that described in *Sigournea* [10], where the upper sections of the arches were formed by unossified Meckelian cartilage, making the fenestrae partly exo- and partly endomeckelian. As in *Sigournea*, the tops of the arches in the jaw are “either flat or rugose” ([10], p.724) and appear to be finished bone, indicating that they are not broken off but rather sutured with an unossified element.

Prior to description of *Sigournea*, two basic Meckelian ossification states had been documented, with a transition between the two occurring in early tetrapod evolution [5]. Arches were primitively formed by endochondral Meckelian cartilage as observed in colosteids, with lack of ossification of this cartilage leading to a fossilized appearance as a single large fenestra. The more derived form was full dermal ossification of these arches, as seen in *Megalocephalus* [2]. The condition in *Acanthostega* (“shallow notches” along the ventral edge of the prearticular – [5], p.25) was hypothesized to be intermediate; *Sigournea* however yet better represents the intermediate state, with only the lower portions of the fenestrae showing dermal ossification. Other taxa such as *Whatcheeria*, *Occidens* and *Crassigyrinus* are possibly reinterpretable in this light as showing a state of ossification similar to that of *Sigournea* [10]. The degree of ossification in the Parrsboro jaw may be similar, but the jaw displays other features which are more derived than in *Sigournea* and the whatcheeriids (such as lack of coronoid dentition), and is geologically later than these taxa. This indicates that the “intermediate” morphology of *Sigournea* may have persisted among later tetrapods, or that the level of ossification may have been variable within individual taxa; this would explain some specimens of *Crassigyrinus* having been documented with fully ossified exomeckelian fenestrae, but Ahlberg and Clack ([2], p. 30) noting in one specimen only “the bases of pillars defining” possible Meckelian fenestrae being present, with cartilaginous dorsal portions presumably having completed the fenestrae. Given the possible variation in this feature, and the other more derived traits shown by the jaw, excluding the jaw as an embolomere, baphetid, or basal temnospondyl based on Meckelian ossification is not possible.

The ornamentation pattern of the Parrsboro jaw is consistent with a crownward – possibly baphetid or temnospondyl – placement. Ornament pattern in the Parrsboro jaw is not extensively known, but both sections visible resemble the coarse, fairly regular pit and ridge pattern seen in many temnospondyls and baphetids as well as the Devonian tetrapods *Acanthostega* and *Ichthyostega* [3]. In embolomeres, ornamentation is irregular and separated by non-ornamented patches [3], and in whatcheeriids [6] dermal ornamentation is generally absent. Other patterns are seen within temnospondyls and baphetids, including tuberculate ornamentation in the “aberrant baphetid” *Spathicephalus* [27], but ornamentation is never absent or reduced. The observed ornamentation pattern thus weakens the case for assignment of the jaw as an embolomere and would tend to support the jaw's placement close to or within baphetids or temnospondyls. *Greererpeton* [5] and other colosteids have similar ornamentation, but more derived features of the Parrsboro jaw, such as lack of coronoid dentition, and the morphology of the Meckelian fenestra (a single large opening with no smaller Meckelian foramina in *Greererpeton* versus smaller Meckelian foramina at the ventral margin of a large opening in the Parrsboro jaw), make a colosteid assignment unlikely.

A temnospondyl assignment of the jaw is plausible. As the only other tetrapod remains found in the Parrsboro formation, identification of the jaw as *Dendrerpeton* would appear to be a possibility. The morphology of the Parrsboro jaw, however, does not bear close resemblance to *Dendrerpeton* [28], with the arrangement of infradentaries, prearticular and Meckelian foramina differing significantly, though preservation of the mesial surface in *Dendrerpeton* is poor. Tooth number of *Dendrerpeton* is also slightly higher than that of the Parrsboro jaw (though maxillary-mandibular discrepancy could be expected), and all *Dendrerpeton* specimens are 100 mm or less in skull length [28], meaning the jaw would be the largest *Dendrerpeton* specimen yet described. The Parrsboro jaw's possession of an adsymphysial plate with fang pair would also be relatively unusual for a temnospondyl, though such a plate has been documented in some temnospondyl taxa [29], [30], and loss of the adsymphysial plate probably occurred separately in stem amniotes and stem Lissamphibia [31], because the most basal stem amniotes such as *Pholiderpeton* (Clack pers. obs.) and *Caerorhachis* [25] possess a plate. However, basal temnospondyls do differ from the Parrsboro jaw in the morphology of their adductor fossa and crest, with *Balanerpeton* showing a maximum of convexity of the adductor crest anterior to, rather than directly above, the adductor fossa ([11]; this is polymorphic in *Dendrerpeton*), and both *Balanerpeton* and *Dendrerpeton* lack a mesial flange of the prearticular adjacent to the adductor fossa [11].

Baphetid assignment of the jaw can also not be ruled out. Milner and Lindsay [22] stated that the presence of an adsymphysial plate precluded the Parrsboro jaw from being a temnospondyl, and conjectured the jaw to be a baphetid (a group probably on either the tetrapod [25] or amniote [[32], [31] excluding mandibular characters] stem, though baphetid affinity with temnospondyls cannot be entirely ruled out [33]). As noted above however, temnospondyls have now been documented possessing an adsymphysial plate ([29], [30]), and the assignment of Milner and Lindsay [22] of the jaw to Baphetidae is otherwise based on characters widely shared with temnospondyls such as a fairly regular coarse dermal ornament pattern, lack of coronoid dentition, and tooth number and shape. Tooth shape, size and number do, however, closely resemble the condition in *Baphetes* (with 16 or 17 dentary teeth countable in *Baphetes kirkbyi* – [23]).

The jaw differs from that of the baphetids *Megalocephalus* and *Baphetes kirkbyi* in various respects, although it is more similar to the latter and cannot be excluded from being a different species of *Baphetes*. When compared to *Megalocephalus* as reconstructed both by Ahlberg and Clack [2], Beaumont [23], and Watson ([34] – as “*Orthosaurus*”) – the baphetid in which mesial mandibular morphology is best known – several features distinguish the Parrsboro jaw. One is prearticular shape and form. In *Megalocephalus* the prearticular is largely or completely undenticulated, and sutures extensively with the postsplenial, interrupted by several small Meckelian foramina. The prearticular of the Parrsboro jaw is fully denticulated and displays no evidence of a suture with the postsplenial or involvement in Meckelian foramen formation, though the postsplenial shape indicates that there were a number of small fenestrae. In *Megalocephalus* the splenial, which is undenticulated unlike in one of our two interpretations of the Parrsboro jaw, forms the dorsal margins of the most anterior Meckelian foramina. The dentary dentition of *Megalocephalus* is highly heterodont, with the Parrsboro jaw much more closely approaching the fundamentally homodont dentition of *Baphetes* in this respect. As noted by Milner and Lindsay [22], the only major character which distinguishes the jaw from *Baphetes* is its possession of denticle shagreen. Much of the arrangement of the prearticular and Meckelian foramina in *Baphetes* remains unknown. As denticulation of coronoids (and possibly the prearticular) is known in *Megalocephalus*, the Parrsboro jaw cannot be excluded as a baphetid based on this dissimilarity, though it can be excluded as an example of *Baphetes kirkbyi*, concurring with the later geological age (earliest from Westphalian B – [23] i.e. Bashkirian) of the latter. All known baphetids lack the dorsal curvature above the adductor fossa documented in the Parrsboro jaw, and those for which the mesial surface is known lack a mesial flange formed by the prearticular posterior to the adductor fossa documented in the Parrsboro jaw ([23], [11], [2]).

Overall the Parrsboro jaw can be placed with reasonable confidence as a stem tetrapod higher on the stem than whatcheeriids and colosteids, or as a crown tetrapod on the amniote or temnospondyl stem. The jaw shows an unusually plesiomorphic feature in the form of a large unossified Meckelian bone. This demonstrates the labile nature of Meckelian ossification within early tetrapod evolution, which is seemingly supported by polymorphism in this character within individual taxa. Furthermore, the jaw's possession of this character demonstrates the dangers of using a single character – such as Meckelian ossification – to place taxa phylogenetically. Whilst exact placement of the Parrsboro jaw remains uncertain, the ability, using mandibular characters, to place the jaw roughly on the tetrapod lineage demonstrates the utility of recent work examining early tetrapod mandibular evolution.

## Supporting Information

Video S1 **Video of 3D laser scan of the peel of the anterior of the Parrsboro jaw, NSM 987GH65.1.**
(MP4)

Matrix S1 **Matrix of Clack et al. (2012) including the Parrsboro jaw, scored with denticulated element below anterior coronoid interpreted as prearticular.** Corresponds to “matrix 1” in the text and to Figs. 1B and 2B.
(NEX)

Matrix S2 **Matrix of Clack et al. (2012) including the Parrsboro jaw, scored with denticulated element below anterior coronoid interpreted as splenial.** Corresponds to “matrix 2” in the text and to Figs. 1C and 2C.
(NEX)

Character List S1 **List of characters corresponding to matrices.**
(DOC)
