# Supplementary material for: Redescription and Phylogenetic Analysis of the Mandible of an Enigmatic Pennsylvanian (Late Carboniferous) Tetrapod from Nova Scotia, and the Lability of Meckelian Jaw Ossification
Source: PLoS One. 2014 Oct 7;9(10):e109717. doi: 10.1371/journal.pone.0109717 (PMC4188710; doi:10.1371/journal.pone.0109717)
Supplement: Character List S1 — List of characters corresponding to matrices. (DOC) [file pone.0109717.s004.doc]

### Character list S1

skull roof, palate

1. Anterior tectal/septomaxilla: anterior tectal (external bone, dorsal to nostril): = 0, septomaxilla (external or internal bone, posterior to nostril) = 1, absent = 2
2. Ectopterygoid /palatine exposure: more or less confined to tooth row = 0, broad mesial exposure additional to tooth row = 1
3. Ectopterygoid as long or longer than palatines: yes, = 0, no = 1
4. Ectopterygoid reaches subtemporal fossa: no = 0, yes = 1
5. Frontal: absent = 0, present = 1
6. Intertemporal: present = 0, absent = 1
7. Jugal: does not extend anterior to orbit = 0, extends anterior to orbit = 1
8. Lacrimal: contributes to orbital margin = 0, excluded from margin = 1
9. Lateral rostral present: yes = 0, no = 1
10. Maxilla makes interdigitating suture with vomer: no = 0, yes = 1
11. Maxilla external contact with premaxilla: narrow contact point not interdigitated = 0, interdigitating suture = 1
12. Maxilla extends behind level of posterior margin of orbit: yes = 0, no = 1
13. Median rostral: single = 0, paired = 1, absent = 2
14. Opercular: present = 0, absent = 1
15. Prefrontal: twice as long as broad, or less = 0, three times as long as broad = 1
16. Prefrontal: transverse anterior suture with tectal = 0, tapers to point anteriorly = 1
17. Preopercular: present = 0, absent = 1
18. Pterygoids separate in midline = 0, meet in midline anterior to cultriform process = 1
19. Pterygoid quadrate ramus margin in subtemporal fossa: concave = 0, with some convex component = 1
20. Vomers separated by parasphenoid > half length: yes = 0, no = 1
21. Vomers excluded from margin of interpterygoid vacuity: yes = 0, no = 1
22. Vomers as broad as long or broader = 0, about twice as long as broad or longer = 1

braincase

1. Basipterygoid process: not strongly projecting with concave anterior face = 0, strongly projecting with flat anterior face = 1
2. Ethmoid: fully ossified = 0, partly or wholly unossified = 1
3. Hypophysial region: solid side wall pierced by small foramina for pituitary vein and other vessels = 0, single large foramen = 1
4. Otic capsule: lateral commissure bearing hyomandibular facets: present = 0, absent = 1
5. Parasphenoid: does not overlap basioccipital = 0, overlaps basioccipital =1
6. Parasphenoid: denticulated field: present = 0, absent = 1
7. Sphenoid: fully ossified, terminating posteriorly in intracranial joint or fused to otoccipital = 0, separated from otoccipital by unossified gap = 1

palatal dentition

1. Ectopterygoid fang pairs: present = 0, absent = 1
2. Ectopterygoid row (3+) of smaller teeth: present = 0, absent = 1
3. Ectopterygoid / palatine shagreen field: absent = 0, present = 1
4. Maxilla tooth number: > 40 = 0, 30-40 = 1, < 30 = 2
5. Palatine row of smaller teeth: present = 0, absent = 1
6. Pterygoid shagreen: dense = 0, a few discontinuous patches or absent = 1
7. Premaxillary tooth proportions: all approximately same size = 0, posteriormost teeth at least twice height of anteriormost teeth = 1
8. Vomerine fang pairs: present = 0, absent = 1
9. Vomerine fang pairs noticeably smaller than other palatal fang pairs: no = 0, yes = 1
10. Vomer anterior wall forming posterior margin of palatal fossa bears tooth row meeting in midline: yes = 0, no = 1
11. Vomerine row of small teeth : present = 0, absent = 1
12. Vomerine shagreen field: absent = 0, present = 1

lower jaw

1. Adductor fossa faces dorsally = 0, mesially = 1
2. Adductor crest: absent = 0, peak anterior to adductor fossa, dorsal margin of fossa concave = 1, peak above anterior part of adductor fossa, dorsal margin of fossa convex = 2
3. Angular – prearticular contact: prearticular contacts angular edge to edge = 0, absent = 1, mesial lamina of angular sutures with prearticular = 2 ?
4. Coronoid (anterior) contacts splenial: no = 0, yes = 1
5. Coronoid (middle) separated from special: yes, by prearticular = 0s, no = 1, yes, by postsplenial = 2
6. Coronoid (middle) contacts postsplenial: no = 0, yes = 1
7. Coronoid (posterior) posterodorsal process: no = 0, yes = 1
8. Coronoid (posterior) posterodorsal process visible in lateral view: no = 0, yes = 1 ?
9. Dentary external to angular + surangular, with chamfered ventral edge and no interdigitations: no = 0, yes = 1 ?
10. Dentary ventral edge: smooth continuous line = 0, abruptly tapering or ‘stepped’ margin = 1 ?
11. Dentary suture with splenial + postsplenial marked by deep furrow: no = 0, yes = 1 ?
12. Mandibular sensory canal: present = 0, absent = 1 ?
13. Mandibular canal exposure: entirely enclosed, opens through lines of pores = 0, mostly enclosed, short sections of open grooves = 1, mostly open grooves, short sections opening through pores = 2, entirely open = 3 ?
14. Mandible: oral sulcus/surangular pit line: present = 0, absent = 1 ?
15. Meckelian bone floors precoronoid fossa: yes = 0, no = 1 ?
16. Meckelian bone ossified in middle part of jaw: yes = 0, little or no ossification = 1
17. Meckelian bone exposure in middle part of jaw, depth much less than prearticular, 0 depth similar to prearticular = 1 N/A
18. Meckelian foramina/ fenestrae, dorsal margins formed by; Meckelian bone = 0, prearticular = 1, infradentary = 2
19. Meckelian foramina/ fenestrae, height: much lower than adjacent prearticular = 0, equal to or greater than depth of adjacent prearticular = 1 ?
20. Parasymphysial lateral foramen present: no = 0, yes = 1 ?
21. Parasymphysial mesial foramen present: no = 0, yes = 1
22. Postsplenial with mesial lamina: no = 0, yes = 1
23. Postsplenial pit line present: yes = 0, no = 1 ?
24. Postsplenial suture with prearticular present: no = 0, yes but interrupted by Meckelian foramina or fenestrae = 1, uninterrupted suture = 2
25. Prearticular sutures with surangular: no = 0, yes = 1 ?
26. Prearticular sutures with mesial lamina of splenial: no, mesial lamina of splenial absent = 0, yes = 1, no, mesial lamina of splenial separated from prearticular by postsplenial = 2
27. Prearticular with longitudinal ridge below coronoids: no = 0, yes = 1
28. Prearticular with mesially projecting flange on dorsal edge along posterior border of adductor fossa: no = 0, yes = 1
29. Prearticular centre of radiation of striations: level with posterior end of posterior coronoid = 0, level with middle of adductor fossa = 1, level with posterior end of adductor fossa = 2 ?
30. Splenial has free ventral flange: yes = 0, no = 1 ?
31. Splenial, rearmost extension of mesial lamina: closer to anterior end of jaw than to adductor fossa = 0, equidistant = 1, closer to anterior margin of adductor fossa than to the anterior end of the jaw = 2 ?

lower jaw dentition

1. Coronoids: at least one has fang pair recognisable because at least twice the height of coronoid teeth: yes = 0, no = 1
2. Coronoids: at least one has fangs recognisable because noticeably mesial to vertical lamina of bone and to all other teeth: yes = 0, no = 1
3. Coronoids: at least one has organised tooth row: yes = 0, no =1
4. Coronoids: at least one carries shagreen: no = 0, yes = 1
5. Coronoids: size of teeth (excluding fangs) on anterior and middle coronoids relative to dentary tooth size: about the same = 0, half height or less = 1 ?
6. Dentary tooth row: homodont = 0, markedly heterodont = 1
7. Dentary with parasymphysial fangs internal to marginal tooth row: yes = 0, no = 1
8. Dentary teeth: same size as maxillary teeth = 0, larger than maxillary teeth = 1, smaller than maxillary teeth = 2 ?
9. Dentary with a row of very small teeth or denticles lateral to tooth row: yes = 0, no = 1
10. Parasymphysial tooth plate: present = 0, absent = 1
11. Parasymphysial plate dentition: shagreen or irregular tooth field = 0, organised dentition aligned parallel to jaw margin = 1, no dentition = 2
12. Parasymphsial plate has fang pair: no = 0, yes = 1.
13. Parasymphysial plate has tooth row: no = 0, short tooth row, separated from coronoid tooth row by diastema = 1, long tooth row reaching coronoid = 2
14. Prearticular shagreen field, distribution: gradually decreasing from dorsal to ventral = 0, well defined dorsal longitudinal band = 1, scattered patches or absent = 2, fully denticulated = 3

# general skull characters

1. Anterior palatal fenestra: single = 0, double = 1, absent = 2
2. Dorsal fontanelle on snout: absent = 0, present = 1
3. Interpterygoid vacuities: absent = 0, at least 2 x longer than wide = 1, < 2 x longer than wide = 2
4. Intracranial joint: present in dermal skull roof = 0, absent = 1
5. Nature of dermal ornament: tuberculate = 0, fairly regular pit and ridge = 1, irregular = 2, absent or almost absent = 3
6. Nature of ornament: ‘starbursts’ of radiating ornament on at least some bones: no = 0, yes = 1

postcranium

1. Anocleithrum: oblong with distinct anterior overlap area = 0, drop-shaped with no anterior overlap area = 1, absent = 2
2. Cleithrum: ornamented = 0, not ornamented = 1
3. Cleithrum, postbranchial lamina: present = 0, absent = 1
4. Digits: absent = 0, present = 1
5. Humerus: narrow tapering entepicondyle = 0, square or parallelogram-shaped entepicondyle = 1
6. Ilium, iliac canal: absent = 0, present = 1
7. Ilium, posterior process: oriented posterodorsally = 0, oriented approximately horizontally posteriorly = 1
8. Interclavicle: small and concealed or absent = 0, large and exposed = 1
9. Interclavicle shape: ovoid = 0, kite-shaped = 1, with posterior stalk = 2
10. Lepidotrichia in paired appendages: present = 0, absent = 1
11. Posttemporal + supracleithrum: present = 0, absent = 1
12. Radius and ulna: radius much longer than ulna = 0, approximately equal length = 1
13. Ribs, trunk: no longer than diameter of intercentrum = 0, longer = 1
14. Ribs, trunk: all straight = 0, at least some curving ventrally = 1
15. Ribs, trunk: all cylindrical = 0, some or all bear flanges from posterior margin which narrow distally = 1, some or all flare distally = 2
16. Scapular blade: absent = 0, small with narrow top = 1, large with broad top = 2
17. Scapulocoracoid: small and tripodal = 0, large plate pierced by large coracoid foramen = 1, very large plate without large coracoid foramen = 2
18. Subscapular fossa: broad and shallow = 0, deeply impressed posteriorly = 1
19. Squamation: complete body covering of scales, all similar = 0, ventral armour of gastralia = 1

new characters

1. Pectoral process: absent = 0, present = 1.
2. Proximal limb of oblique ridge: present, separated from anterior margin of humerus by prepectoral space = 0, absent, replaced by deltopectoral crest = 1.
3. Latissimus dorsi attachment: diffuse ridged area = 0, distinct process = 1.
4. Foramina piercing oblique ventral ridge: many = 0, one moderately large foramen in addition to entepicondylar foramen = 1, entepicondylar foramen is the only large opening, other foramina are tiny pinpricks or absent = 2
